# Transformation of Lipid Bodies Related to Hydrocarbon Accumulation in a Green Alga, *Botryococcus braunii* (Race B)

**DOI:** 10.1371/journal.pone.0081626

**Published:** 2013-12-05

**Authors:** Reiko Suzuki, Naoko Ito, Yuki Uno, Ichiro Nishii, Satoshi Kagiwada, Sigeru Okada, Tetsuko Noguchi

**Affiliations:** 1 Department of Biological Sciences, Nara Women's University, Nara, Nara, Japan; 2 Temasek Life Sciences Laboratory, Singapore, Singapore; 3 Department of Biological Sciences, National University of Singapore, Singapore, Singapore; 4 Department of Aquatic Biosciences, the University of Tokyo, Bunkyo, Tokyo, Japan; 5 Japan Science and Technology Agency-CREST, Chiyoda, Tokyo, Japan; Argonne National Laboratory, United States of America

## Abstract

The colonial microalga *Botryococcus braunii* accumulates large quantities of hydrocarbons mainly in the extracellular space; most other oleaginous microalgae store lipids in the cytoplasm. *Botryococcus braunii* is classified into three principal races (A, B, and L) based on the types of hydrocarbons. Race B has attracted the most attention as an alternative to petroleum by its higher hydrocarbon contents than the other races and its hydrocarbon components, botryococcenes and methylsqualenes, both can be readily converted into biofuels. We studied race B using fluorescence and electron microscopy, and clarify the stage when extracellular hydrocarbon accumulation occurs during the cell cycle, in a correlation with the behavior and structural changes of the lipid bodies and discussed development of the algal colony. New accumulation of lipids on the cell surface occurred after cell division in the basolateral region of daughter cells. While lipid bodies were observed throughout the cell cycle, their size and inclusions were dynamically changing. When cells began dividing, the lipid bodies increased in size and inclusions until the extracellular accumulation of lipids started. Most of the lipids disappeared from the cytoplasm concomitant with the extracellular accumulation, and then reformed. We therefore hypothesize that lipid bodies produced during the growth of *B. braunii* are related to lipid secretion. New lipids secreted at the cell surface formed layers of oil droplets, to a maximum depth of six layers, and fused to form flattened, continuous sheets. The sheets that combined a pair of daughter cells remained during successive cellular divisions and the colony increased in size with increasing number of cells.

## Introduction

To combat global warming by minimizing the carbon dioxide emitted by the combustion of fossil fuels, alternative energy sources, including biofuels, are receiving increasing attention from researchers. Biofuels include ethanol and methane generated by fermentation of plant biomass, and lipids such as triacylglycerols produced by plants. Aerobic photosynthetic microorganisms can also produce substantial amounts of lipids [1], and the potential productivity of oleaginous microalgae per unit area can be 8 to 24 times that of the best land plants [2].

Among the oleaginous microalgae, the colonial green alga *Botryococcus braunii* is interesting because it accumulates large quantities of hydrocarbons other than triacylglycerols [3]. Moreover, most of the hydrocarbons produced by *B. braunii* accumulate in the extracellular space, whereas other microalgae that have been studied, including oleaginous species in which active lipid production is induced by environmental stress [4]–[6], store lipids in their cytoplasm. *B. braunii* is classified into three principal races (A, B, and L) based on the types of hydrocarbons they synthesize [7] [8]. Race A produces alkadienes and alkatrienes derived from fatty acids [9]; race B produces triterpenoids known as botryococcenes and methylsqualenes [10] [11]; and race L produces a tetraterpenoid known as lycopadiene [12]. Race B has attracted the most attention as an alternative to petroleum because it generally has a higher content of hydrocarbons than the other races [7], and its hydrocarbons (botryococcenes and methylsqualenes) are expected to be readily converted into biofuels [13]. Since 1984, the biosynthetic pathways to produce botryococcenes and methylsqualenes have been studied, and several important enzymes that contribute to the production of these triterpenoid hydrocarbons have been identified [14]–[18].

Despite the increased chemical and biochemical information on the hydrocarbons produced by this alga, the information where these hydrocarbons are formed in cells and how they are secreted into the extracellular matrix has not been well characterized. Because the hydrocarbons produced by all races of *B. braunii* accumulate mainly in the extracellular matrix, most of them can be extracted by soaking the dried algae in a non-polar organic solvent such as *n*-hexane that does not permeate the cells. Hydrocarbons obtained in this way are defined as “external hydrocarbons” [19]. After the external hydrocarbons have been recovered, a small amount of hydrocarbons that are defined as “internal hydrocarbons” can be obtained by extraction with a mixture of chloroform and methanol that can reach inside the cells [19].

When race A of *B. braunii* was fed with radio-labeled precursors for hydrocarbon biosynthesis, radioactivities did not shift from the internal hydrocarbons to the external hydrocarbons [20]. On the other hand, similar experiments carried out using race B revealed the radioactivity clearly shifted from the internal fraction to the external one [10]. Therefore, the sites of hydrocarbon biosynthesis and the mechanisms of hydrocarbon secretion may differ between races A and B. Considering the practical application of *B. braunii* for biofuel production, it is very important to develop a system to recover hydrocarbons from the alga with a low input of energy, and several papers on effective hydrocarbon recovery have been published [21]–[24].

In this context, information on the structure of the algal colonies, the sites of hydrocarbon production, and the alga's hydrocarbon secretion system would be critical for understanding the mechanism. Nevertheless, the major ultrastructural studies with electron microscopy were limited to the period from 1978 to 1984 [19] [25]–[27]. In the case of *B. braunii*, the use of conventional chemical fixation for electron microscopy failed to show the fine ultrastructure of the cells, especially the cytoplasm, probably because the large amount of lipid that accumulated around cell surfaces interfered with the penetration of fixatives and embedding resin. We have overcome this difficulty by using a rapid freezing and freeze-substitution method for electron microscopic analysis on race A and clarified that active hydrocarbon synthesis occurred just after septum formation during cell division [28]. Lipid bodies in the cytoplasm were few in interphase cells, increased in number and size until the first lipid accumulation on the cell surface at the cell apex. Most of them disappeared from the cytoplasm concomitant with their main accumulation in the basolateral region on the cell surface [28]. However, the analysis of race B has not been done until a recent paper by Weiss et al. [29]. Their images were obtained by means of a rapid-freezing method—quick-freeze deep-etch electron microscopy—and clarified the colony organization. In fact, it is more difficult to obtain clear images by means of thin section for electron microscopy, probably because this race accumulates more hydrocarbons than race A on the cell surface.

Here, we have therefore used fluorescence microscopy and transmission electron microscopy to obtain fine images showing colonial cells with extracellular hydrocarbons and lipid bodies. Then, the behavior and structural changes of the lipid bodies during the cell cycle were analyzed in detail and strongly suggests that they are rather directly involved in secretion of hydrocarbons. We also examined effects of treatment of *n*-hexane and cellulase on ultrastructure of the extracellular matrix to know the contribution of the hydrocarbons and the polysaccharides to the colony organization of race B. Overall, the characteristics of lipid bodies and hydrocarbon secretion in race B were very similar to that of race A, but we also detected quantitative differences of them that could account for larger production of hydrocarbons in race B.

## Materials and Methods

### Strains and culture conditions

*Botryococcus braunii* race B Berkeley (Showa) was cultured in modified Chu13 medium at 22°C on shaker at 80 rpm. They were illuminated for 13 h per day with fluorescence light at a photon flux density of 50 µmol ⋅ m^–2^ ⋅ sec^–1^. One-month-old cell cultures were transferred to a fresh culture medium 1 h after illumination to induce cell division.

### Cell collection

Cell cultures were first condensed by filtration on 10 µm nylon-mesh on a magnetic filter funnel (PALL), and then the condensed cell culture was centrifuged using Viva-spin ultrafiltration columns (GE Healthcare Life Sciences) at 3,000 rpm for 2 min to remove excess media. The cells on the filter were used for experiments or electron microscopy.

### Light and fluorescence microscopy

For double staining with Nile red and neutral red, living cells were soaked in 0.05% neutral red dissolved in culture medium for 20 min. Then 1/100 volume of 1 mM Nile red solution (stock solution dissolved in dimethyl sulfoxide) was added, and the cells stained for another 10 min. The cells were mounted on a glass slide slightly pressed with a coverslip observed by using light (neutral red) and fluorescence (Nile red) microscope, Olympus BX51. For morphometry analysis, the cover slip was pressed a little more to obtain most of lipid bodies/vacuoles in a focal plane.

Cells with fine staining of lipid bodies (LBs, yellow fluorescence) and vacuoles (red) on magnified photographs at x100,000 were selected for measurement. Fifty cells at each stage 1, 2, 4 and 5 and fifty pairs of daughter cells at stage 3 were statistically evaluated by Scheffe's multiple comparison test (IBM SPSS statistics Version 21).

### Treatment with *n*-hexane and degrading enzymes for cell wall

Cell cultures were collected on 10 µm nylon mesh filter. The filter with cells was placed on paper wipers (Kimwipe) to absorb excess culture medium, and then placed in a conical Fernbach flask (100 ml), added 5 ml *n*-hexane, and then placed upon orbital shaking 80 rpm for 15 min at 22°C. For cellulase treatment, collected cells with or without the *n*-hexane treatment were transferred to test tubes and treated with 8% cellulase (ONOZUKA-RS; Yakult Pharmacheutical) and 2% macerozyme (R200; Yakult Pharmacheutical) for 16 h at 30°C under continuous light.

### Electron microscopy

*B. braunii* cells at different developmental stages were quick-frozen by a high pressure freezing method or by liquid propane. For the high pressure freezing method, concentrated cell cultures were filled into the gold carrier ‘hat’ (1.2 mm in diameter and 200 µm in depth). After removal of extra medium, 1-hexadecene was dropped on the sample to fill empty space. The hat was frozen by Leica-EMPACT2 (Leica Microsystem, Wetzlar) under 210 MPa and transferred in liquid nitrogen. For liquid propane freezing, concentrated cell cultures were attached to formvar films mounted on copper wire loops 8 mm in diameter and were quickly frozen in liquid propane at −190°C. Frozen cells were then transferred to acetone (−85°C) that contained 3% osmium tetroxide and 0.2% uranyl acetate. After ≥48 h at −85°C, the samples were gradually warmed to room temperature, washed with acetone, and embedded in Spurr resin. Sections were stained with lead citrate and examined in a JEM-1230 transmission electron microscope (JEOL). In total, we repeated the fixation 14 times as independent experiments and took photographs by film more than 250 interphase cells, 130 growing cells before septum formation, 130 cells during and just after septum formation, 500 cells accumulating lipids on the surface and 400 mature daughter cells, to obtain representative images for the different stages shown here.

## Results

### Colony organization of *Botryococcus braunii* race B

**Figure 1:**
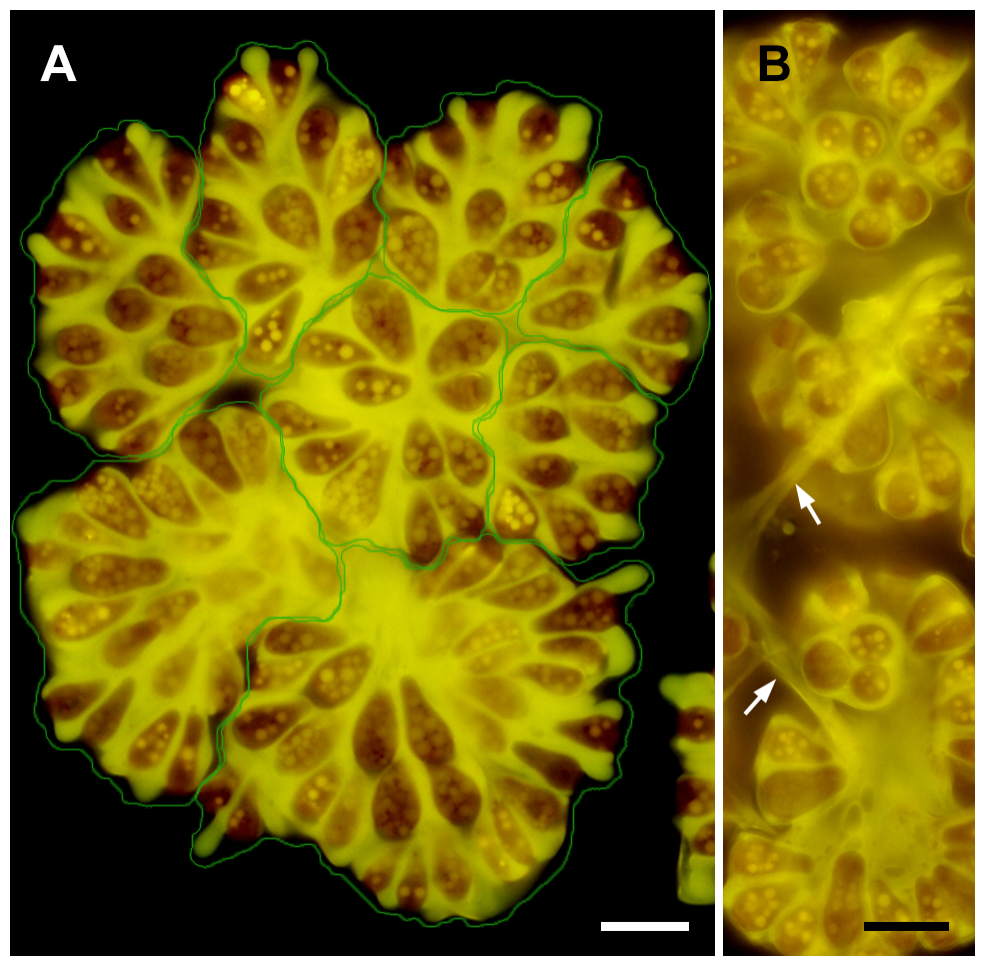
Colony organization of *Botryococcus braunii* race B by fluorescence microscopy. **A**. A colony consists of 8 sub-colonies (enclosed by green line), in which cells are arranged as like a bouquet. Lipids and extracellular matrix composed of hydrophobic biopolymers are stained with Nile red as yellow. **B**. Refracting threads (white arrow). Scale bars: 10 µm.

*Botryococcus braunii* is a colony-forming green alga with pyriform-shaped cells. To observe the colony organization of race B, colonies were stained with the fluorescence lipophilic dye Nile red, then mounted on glass slides and gently compressed by pressure on a cover slip. A typical *B. braunii* colony comprised several sub-colonies (daughter colonies), and cells within a sub-colony were arranged in the form of a spherical bouquet in which the round cell apex was directed towards the outer surface of the sub-colony (Fig. 1A). Lipids that were stained with Nile red appeared yellow and accumulated greatly in the intercellular matrix. In Figure 1B, Nile red-positive strands connect the daughter colonies, and these strands may correspond to the strand of refringent material [19] or refracting threads [30] that were previously observed by means of light microscopy.

**Figure 2:**
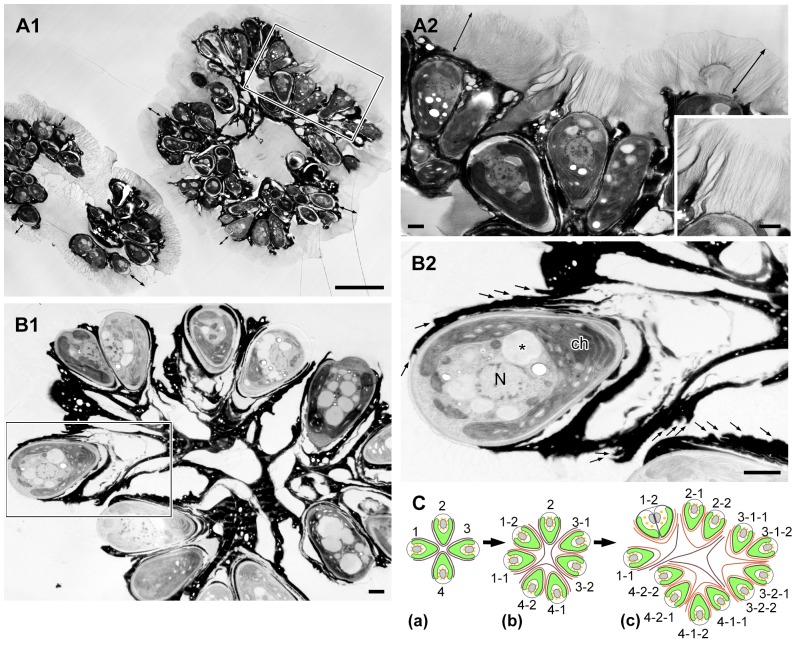
Colony of *B. braunii* race B by electron microscopy. **A**. Sample frozen in liquid propane, **B**. Sample frozen by high-pressure freezing machine. **A1**. Each colony is enclosed by colony sheath (double ended arrow). **A2**. Enlargement of the rectangle in A1. Colony sheath is composed of fibrils stretching from the apical region of each cell and the upper edge of the electron-dense intercellular matrix. Inset is the enlargement of a part of A2. **B1**. Each cell is covered with 3–6 electron-dense thin layers in the basolateral region. These layers are appeared to be holding the colonial cells together. **B2**. Enlargement of the rectangle in B1 (small arrow indicates each electron-dense thin layer). **C**. A speculative genealogic relationship among the cells in the colony shown in B1. ch, chloroplast; N, nucleus; *, lipid body. Scale bars in A1 and A2–B2: 10 µm and 1 µm, respectively.

**Figure 5:**
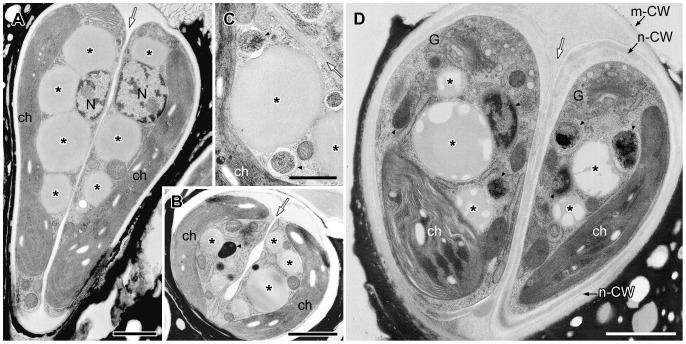
EM analysis of lipid bodies after septum formation to just completing cell wall formation. **A**. Longitudinal section of dividing cells just after septum formation but before cell wall formation. **B**. Cross section of septum developing cell. **C**. Lipid bodies contact to electron-dense bodies enclosed unit membrane. **D**. A pair of daughter cells during cell wall formation. Note that all lipid bodies in the earlier stage (A) are filled with a similar electron-dense material but in D they became patchy with emergence of electron-dense bodies. ch, chloroplast; G, Golgi body; m-CW, mother cell wall; N, nucleus; n-CW, new cell wall; arrowhead, electron-dense body enclosed by unit membrane; white arrow, septum; * lipid body; arrowhead, electron-dense body enclosed by unit membrane. Scale bars in A,B,D and C: 1 µm and 0.5 µm, respectively.

**Figure 6:**
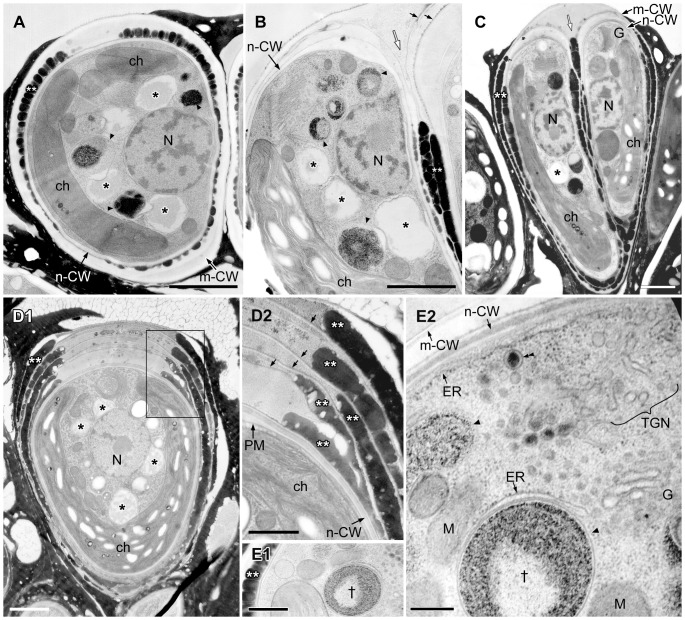
EM analysis of lipid bodies in a pair of daughter cells accumulating oil droplets. Between a new cell wall and a mother cell wall at the basolateral region, oil droplets arrange side by side and form 1-6 layers; one layer in A, E, two layers in B, C, and five layers in D are observed. **A–C**. Lipid bodies contact to electron-dense bodies enclosed unit membrane. **D**. There is a thin layer between oil droplets layers. **D2**. Enlargement of the rectangle in D1. **E**. Cell accumulates one oil droplet layer. **E2**. Enlargement of a part of E1, in which electron-dense body enclosed by unit membrane marked with † is same marked one in E1. The rER lacks ribosomes on the surface facing the plasma membrane or electron dense body. ch, chloroplast; ER, endoplasmic reticulum; G, Golgi body; m-CW, mother cell wall; M, mitochondrion; N, nucleus; n-CW, new cell wall; PM, plasma membrane; TGN, *trans*-Golgi network; * lipid body; **, oil droplet on the cell surface; small arrow, thin layer between oil droplets layers; white arrow, septum; arrowhead, electron-dense body enclosed by unit membrane; double arrowheads, Golgi vesicle. Scale bars in A-D1, D2-E1 and E2: 1 µm, 0.5 µm, and 0.2 µm, respectively.

When algal colonies were frozen in liquid propane before electron microscopy, we observed a zone of low electron density enclosing each colony in thick sections (Fig. 2A1, double end arrow). At higher magnification, the zone was composed of many fibrils 4 to 7 µm in length that stretched from the cell apex and the upper edge of the electron-dense materials that accumulated in the intercellular matrix (Fig. 2A2). We consider this fibrillar zone to be similar to the fibrillar sheath or colony sheath that Weiss et al. [29] reported using quick-freeze deep-etch electron microscopy. Weiss et al. [29] also clarified that the sheath was composed of arabinose-galactose polysaccharides. The center of the colony was filled with amorphous materials that had an electron density similar to that of the fibrillar sheath (Fig. 2A1). Interestingly, the fibrillar sheath was less visible in sections of samples frozen under high pressure (210 MPa) for electron microscopy, because its electron density became lower (in all electron microscopy images except Fig. 2A, 5A, 6D).

Typically, each colonial cell was covered by three to six electron-dense thin layers at the basolateral region that occupied 70 to 90% of the cell surface (Fig. 2B2, small arrow), and these layers held together the neighboring cells to form a colony (Fig. 2B1). These electron-dense layers correspond to the “outer wall layers” [19], “outer cell walls” [26], and “external walls or external wall layers” [27]. We noticed that neighboring pairs of cells appeared to share an outer set of dense layers, suggesting that the layers had formed before the cells divided. Based on this morphology, we hypothesize that each cell divided to produce a lineage that resulted in the sub-colonies shown in Figure 2B1, following the mechanism shown in Figure 2C.

### Behavior of lipid bodies and vacuoles during the cell cycle

**Figure 3:**
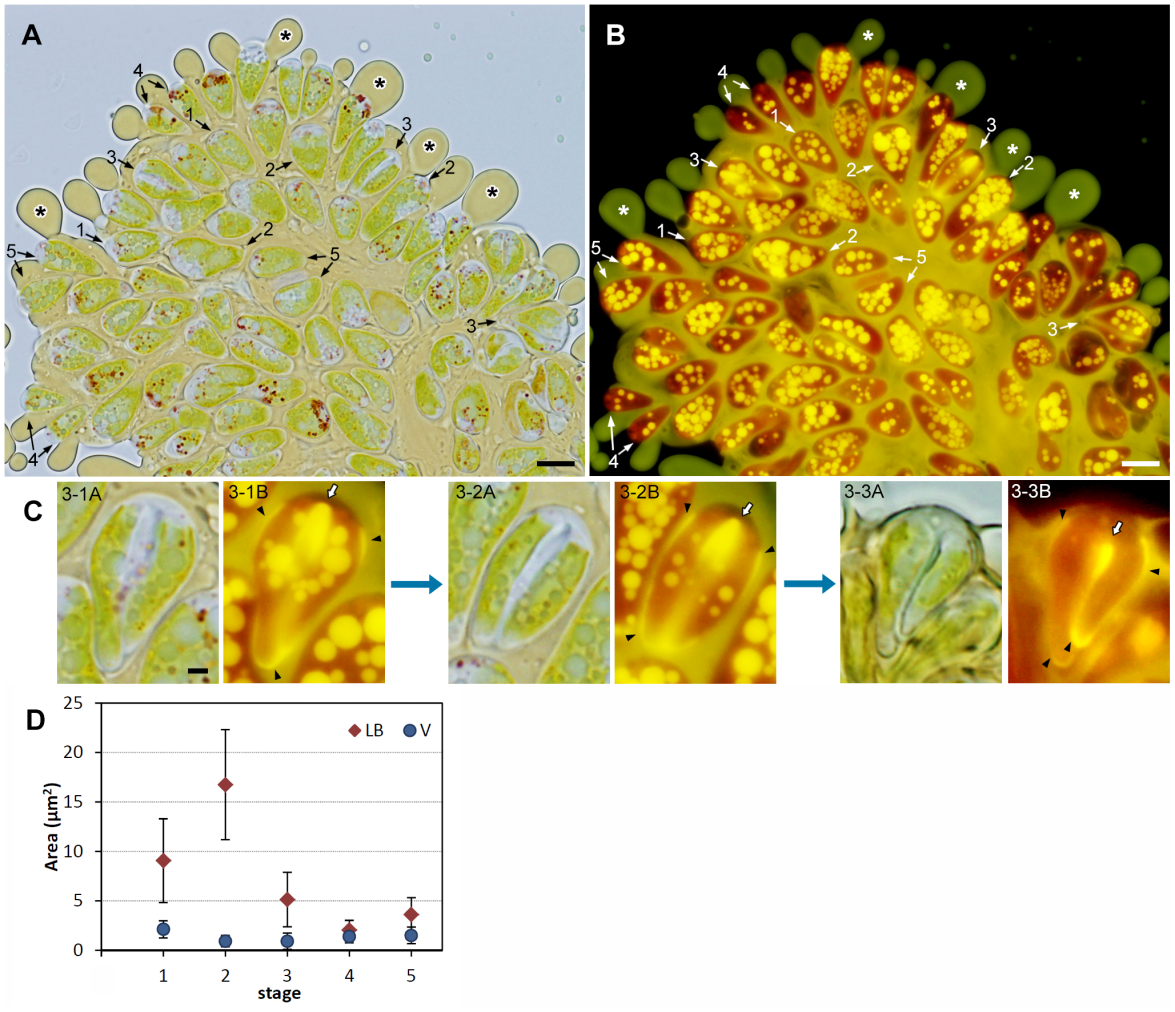
Colony double-stained with Nile red and neutral red. **A**. Neutral red, bright-field microscopy. **B**. Nile red, fluorescence microscopy. Numbers 1–5 with arrows are indicating representative cells or cell pairs at the specific stages of cell cycle as 1; interphase cell, 2; growing cell ∼ cells just after septum formation, 3; cells accumulating lipids between the septum and on the cell surface at basolateral region, 4; a pair of immature daughter cells whose cell area is <2/3 of interphase cell, and 5; A pair of mature daughter cells whose cell area is ≥2/3 of interphase cell. *, exuded lipids from the extracellular matrix. **C**. Three typical staining patterns seen for cells at the stage 3. **3-1∼3A**. Neutral red, **3-1∼3B**. Nile red. **3-1**. A dividing cell with lipid bodies accumulates lipids at the edge of the septum. **3-2**. A dividing cell with a reduced number of lipid bodies accumulates lipids around the septum. **3-3**. A dividing cell without lipid bodies filled lipids around the septum. Lipids on the cell surface at basolateral region are also accumulated for these cells. Arrowhead, newly accumulating lipids on the cell surface at basolateral region; white arrow, septum. **D**. Changes in the total area of lipid bodies (red diamond) and vacuoles (blue circle) per cell. Stage number is corresponding to the number in **A**/**B**. For each stage, the average area of the lipid bodies (LB) and vacuoles (V) per cell are shown (*n* = 50 for each stage, error bars indicate standard deviation). The total area of lipid bodies per cell differed significantly (*p*<0.01) among the five stages, except for between stage 4 and 5. Scale bars in A, B and C: 10 µm and 2 µm, respectively.

Following the conventional method, we transferred 1-month-old cell cultures into fresh culture medium 1 h before the start of illumination, and about 20% of the cells entered the cleavage stage during the dark period of the following day. To observe the behaviors of the lipid bodies and the vacuoles during the cell cycle, we double-stained the colonies with Nile red and neutral red, mounted them on glass slides, then compressed them firmly by pressure on the cover slip to obtain most of lipid bodies and vacuoles in the same focal plane, which was suitable for two-dimensional morphometry. With this double-staining method, cells clearly showed lipid bodies with yellow fluorescence and vacuoles with red, and revealed liquid lipids accumulating in the extracellular matrix with yellow fluorescence and more lipids exuded from the extracellular matrix around the colonies, which were seen with a yellowish-green fluorescence (* in Fig. 3A,B compare to Fig. 1). In a typical colony, cells at different stages in the cell cycle were observed, and we defined the five stages shown in Figures 3A, B as follows: stage 1, interphase cells; stage 2, growing cells (with increased cell volume) ∼ just after septum formation; stage 3, cells accumulating lipids in the septum (between two daughter cells) and on the surface of the basolateral region of the cells; stage 4, a pair of immature daughter cells whose cell area in the micrograph was <2/3 of the area of a typical interphase cell; and stage 5, a pair of mature daughter cells whose cell area was ≥2/3 of the area of an interphase cell. As shown in Figure 3D, we quantified the total area of lipid bodies and vacuoles per cell in stage 1∼ stage 5 using 2D projections. It clearly demonstrated changes in the sizes (area) of the lipid bodies during the cell cycle. Cells contained lipid bodies and vacuoles throughout the cell cycle. In interphase cells (stage 1), lipid bodies ranged in area from 0 to 3 µm^2^, and there were about 9 per cell (Fig. 3D, stage 1). In growing cells (stage 2), lipid bodies had a similar range of sizes, but there were almost twice as many lipid bodies as in stage 1 (compare Fig. 3D stage 1 and stage 2). In cells accumulating lipids in the septum (stage 3), the lipid bodies were smaller and their number decreased greatly (Fig. 3B, No. 3). In Figure 3C, the process of lipid accumulation in the septum between daughter cells is shown in detail. During early stage (Fig. 3C (3-1B)), a cell accumulating lipids at the upper part (proximal to the cell apex) of the septum (white arrow) still contains lipid bodies of various sizes, whose number has decreased from the previous stage. Towards the middle stage (Fig. 3C (3-2B)), cells can be seen accumulating more lipids at both ends of the septum, with a substantial decrease in the number of lipid bodies. During late stage (Fig. 3C (3-3B)), cells can be seen accumulating lipids throughout the septum, and Nile red–positive lipid bodies are totally lost. Note that these dividing cells also newly accumulate lipids in the basolateral region that occupied 70 to 90% of the cell surface (Fig. 3C (3-1B,2B,3B), arrowhead). These results show that the decrease in the number of lipid bodies during this stage is strongly correlated with the accumulation of lipids on the cell surfaces, in the septum, and in the basolateral region (Fig. 3C,D). In a pair of immature daughter cells (stage 4), a few small lipid bodies reappear (Fig. 3B,No. 4; 3D,stage 4). During cell maturation (stage 5), the lipid bodies increased in number and size (Fig. 3B;No. 5, 3D;stage 5).

The total area of lipid bodies per cell differed significantly (*p*<0.01) among the five stages (Fig. 3D, red diamond), except for a lack of difference between stage 4 and stage 5. In contrast to the lipid bodies, vacuoles stained with neutral red did not change drastically in number or size throughout the cell cycle (Fig. 3D, blue circle).

### Cell ultrastructure during the cell cycle

**Figure 4:**
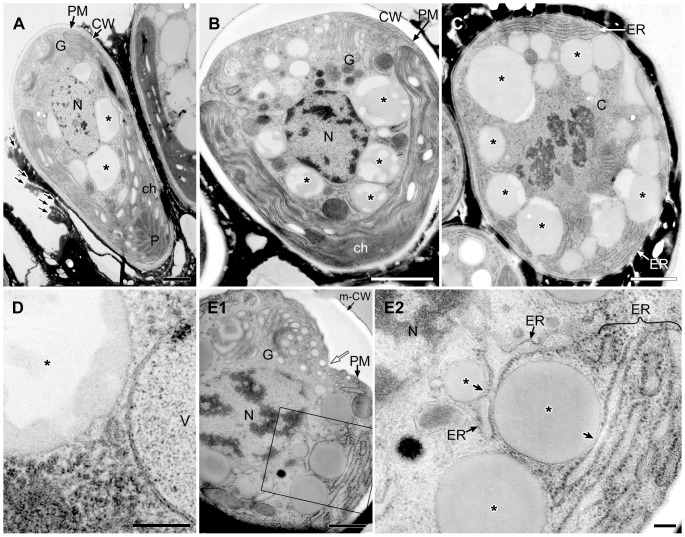
EM analysis of lipid bodies in cells from interphase to early septum formation. **A**. Longitudinal section of interphase cell. The nucleus located at the center and a cup-shaped chloroplast occupies the bottom and side region, large lipid bodies are seen between them. The Golgi apparatus is at cell apex. **B**. Tangential section of early growing cell with swelling extracellular space at the apical region. **C**. Cross section of metaphase cell. **D**. Comparison of limiting membrane between lipid body (lipid monolayer) and vacuole (lipid bilayer). **E**. Tangential section at apical region of cell at early septum formation. **E2**. Enlargement of E1. C, chromosome; ch, chloroplast; CW, cell wall; ER, endoplasmic reticulum; G, Golgi body; N, nucleus; m-CW, mother cell wall; P, pyrenoid; PM, plasma membrane; V, vacuole; * lipid body; thick arrow, contact site between lipid body and the ER; thin black arrow, thin layer; white arrow, septum. Scale bars in A-C,E1 and D,E2: 1 µm, 0.5 µm and 0.1 µm, respectively.

**Figure 7:**
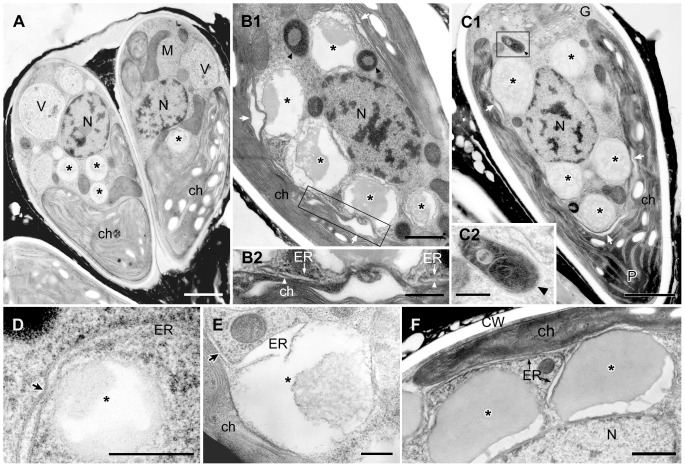
Reformation of lipid bodies during maturation of daughter cells. **A**. Tangential section of a pair of daughter cells. While nuclei are still closely localized to the septum region, the cup-shape of chloroplasts is recovering. A few lipid bodies are seen but still smaller. **B**. **C**. Tangential sections of cells liberated from mother cell wall have recovered chloroplast. Both number and size of lipid bodies increase. They often have a contact site/region to chloroplast, where chloroplast envelope bud to lipid bodies (white arrow). **B2**. Enlargement of the rectangle in B1. rER lost ribosomes from the surface facing toward the chloroplast (white arrowhead). **C1**. Inclusions of lipid bodies are different from those in B1. **C2**. Enlargement of the rectangle in C1 shows an electron dense body with unit membrane contains electron transparent thin layers. **D**. rER contacting with the lipid body lacks ribosomes at the contact site. **E**. **F**. rER near the chloroplast envelope reach to the surface of lipid bodies and had lost ribosomes from the surface facing toward them. ch, chloroplast; CW, cell wall; ER, endoplasmic reticulum; G, Golgi body; N, nucleus; V, vacuole; * lipid body; thick black arrow, ER lacking ribosomes from the surface facing toward the lipid body or the chloroplast. Scale bars in A,B1,C1, B2,F and C2,D,E: 1 µm, 0.5 µm and 0.2 µm, respectively.

We examined the corresponding changes in lipid bodies and vacuoles throughout the cell cycle at the ultrastructural level. Figure 4A shows a typical cell at interphase, with a nucleus (N) located at the center, a cup-shaped chloroplast (ch) possessing a large pyrenoid (P) in the basolateral region of the cell, and a Golgi apparatus (G) at the cell's apex. The space between the nucleus and the chloroplast contained lipid bodies (*) and vacuoles. The endoplasmic reticulum (ER), which is generally believed to occur as one fenestrated sheet per cell, was prominent at the cell apex near the Golgi apparatus and near the lipid bodies, the plasma membrane (PM), and the chloroplast envelope. These cell organelles generally remained in the same position within the cell throughout the cell cycle. The cell wall (CW) was a layer with a uniform thickness that completely surrounded the plasma membrane and was covered with electron-dense thin layers (Fig. 4A, small arrow) in the basolateral region. The mitosis of *B. braunii* progressed in the following order. First, a space between the mother cell's wall and the plasma membrane appeared only at the cell apex (Fig. 4B). Next, binary fission of the cup-shaped chloroplast occurred (not shown). This was followed by nuclear division (Fig. 4C), septum formation during division of the protoplast (Fig. 5A,B), formation of a new cell wall around the daughter cells (Fig. 5C,D), and lipid accumulation on the cell surface in the basolateral region (Fig. 6A–E). Finally, the chloroplast recovers its cup shape (Fig. 7A–C). In the next section, we will describe the ultrastructural morphology of the lipid bodies during these events.

### Ultrastructure of the lipid bodies and related cell organelles during the cell cycle

Our fluorescence microscopy data revealed that lipid bodies changed drastically during two specific stages of the cell cycle of *B. braunii* race B. The first big change, in which the number and size of the lipid bodies increased, was from the growing stage to just after septum formation. The second change, in which the lipid bodies were reformed, was during the maturation of the daughter cells after new accumulation of lipids at the cell surface. Therefore, we examined the ultrastructural changes in the lipid bodies and their association with other organelles; the ER, chloroplast and electron dense body enclosed by a unit membrane. Particles that lacked a unit membrane (the lipid bi-layer) but that contained a lipid mono-layer were identified as lipid bodies (* in Fig. 4D), because their size, location within the cell, and behavior were similar to those of the lipid bodies stained with Nile red in Figure 3.

First, we noticed changes in the contents of the lipid bodies as the cells began septum formation. In the interphase cells, the contents of lipid bodies between the nucleus and the chloroplast consisted of both electron-transparent and electron-dense regions (Fig. 4A), and the proportion of the total area occupied by the electron-transparent areas varied. In cells during the early stage of cell growth before nuclear division (Fig. 4B), the electron-dense contents of the lipid bodies obviously increased; more than half of the contents consisted of electron-dense regions. During mitosis, the lipid bodies increased in number and their electron-dense inclusions gradually filled most of the lipid body (Fig. 4C,E). At this stage, the swollen ER was prominently visible in the upper region near the Golgi apparatus. Lipid bodies often attached to the rough endoplasmic reticulum (rER), whose contact site was narrow, and the rER had lost ribosomes near the contact site (Fig. 4E2, arrow). In cells just after septum formation but before cell wall formation (Fig. 5A), the lipid bodies contained no electron-transparent regions, they had the clear outline expected for a lipid mono-layer, and they occupied most of the space between the nucleus and the chloroplast. However, during cell wall formation around the daughter protoplasts (Fig. 5D), the lipid bodies maintained their clear outline, but electron-transparent regions appeared again.

After cell wall formation but before recovery of the chloroplast, round electron-dense droplets appeared on the cell surface; these appeared between the old mother cell wall and the newly synthesized daughter cell walls, in the basolateral region of the cells (Fig. 6A–E, white **). Lipid bodies decreased in number and also developed lower electron densities (Fig. 6A–D, *). These transmission electron micrograph images correspond to the pairs of cells stained with Nile red in Figure 3C, therefore these round electron dense-droplets on the cell surface in Figure 6 appear to be oil droplets that recently accumulated on the cell surface. The oil droplets on the cell surface formed a layer consistent with the side-by-side arrangement of the oil droplets, and we observed one to six layers. The pair of daughter cells, with a single oil droplet layer (Fig. 6A) or two layers (Fig. 6B), were clearly visible during the process of lipid accumulation, forming multiple layers of lipid droplets (Fig. 6D). Around the cells that were covered by several layers of lipid droplets, another thin layer formed between the lipid droplet layers (Fig. 6B–D), and it enclosed not only the basolateral region, but rather the whole cell. As these oil droplets increased in number, the lipid bodies decreased in number in the cytoplasm, and their inclusion became more electron-transparent. Often, they were attached to spherical bodies that were enclosed by a unit membrane and included electron-dense materials and sometimes electron transparent thin layers (Fig. 6A,B,E2; 7C2, arrowhead). They had appeared during the early growing stage before nuclear division (Fig. 4B). The cortical rER and the rER covering the electron-dense bodies lost ribosomes from the surface facing the plasma membrane or the electron-dense bodies (Fig. 6E2).

After completion of the accumulation of lipids at the cell surface, daughter cells entered the maturation stage (Fig. 7). The mother cell wall decomposed at the cell apex, and the electron-dense layers outside the cell wall were no longer granular, but instead appeared to be flat-oil layer, probably as a result of the fusion of oil droplets (Fig. 7A). Then, the chloroplast recovered in volume and became cup-shaped again, and the nucleus migrated from the septum side to the cell center (Fig. 7B,C). Lipid bodies reappeared, and their electron-dense regions seemed to increase concomitant with cell maturation, although the inclusions varied among images (compare Fig. 7C1 to others in Fig. 7). The maturing lipid bodies often contacted the chloroplast envelope (Fig. 7B,C1) or the rER (Fig.7D–F). At the contact sites, we observed three unique ultrastructures: First, the chloroplast envelope and a few thylakoids protruded into the maturing lipid bodies (Fig. 7B1). The protruded chloroplast envelope contacted the electron-dense inclusions in the lipid bodies, but not the electron-transparent inclusions (Fig. 7B2). Second, a transparent area appeared in chloroplast near the contact sites, whose electron density was lower than in other parts of the stroma except for starch granules (Fig. 7B,C1). Third, at the contact site between the lipid bodies and the rER, the rER lacked ribosomes (Fig. 7D,E). Sometimes, the rER near the chloroplast envelope reached the surface of the lipid bodies, and had lost ribosomes from the surface facing the chloroplast envelope and lipid bodies (Fig. 7E,F).

### Treatment with *n*-hexane and cell wall-degrading enzymes

To obtain ultrastructural information on certain chemical composition of extracellular matrix, we treated colonies with reagents extracting lipids (*n*-hexane) and/or enzymes degrading polysaccharides (cellulase RS and macerozyme).

**Figure 8:**
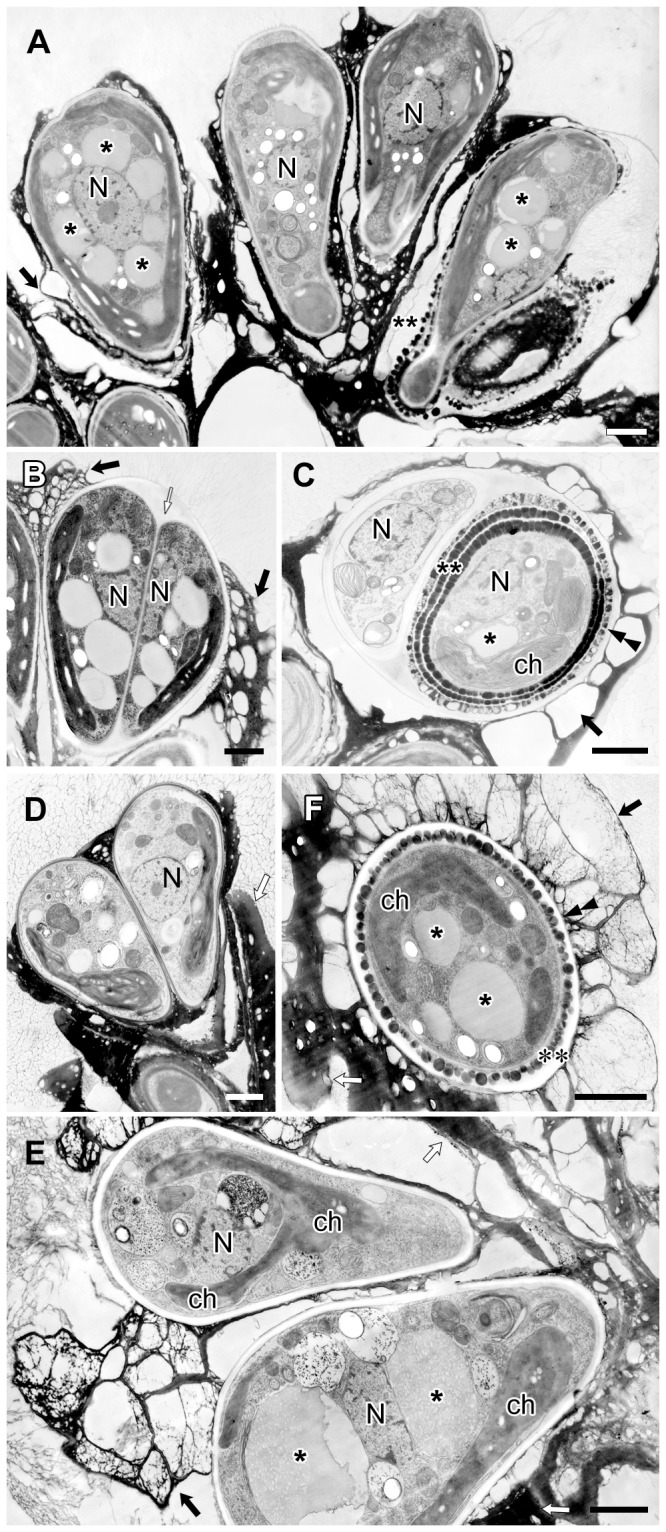
EM analysis of cells treated with reagents degrading extracellular matrix. **A–C**. Treatment with *n-*hexane for 15 min. **D**. Treatment with cellulase RS and macerozyme for 16h. **E**. **F**. Treatment with *n*-hexane for 15 min followed by cellulase RS and macerozyme treatment for 16 h. In C and F, newly secreted lipid droplets on the cell surface remains intact, suggesting that the thin layer (double arrowheads) was resistant to these treatments and protected cellular and extracellular structures. ch, chloroplast; N, nucleus; *, lipid body; **, oil droplet on the cell surface; thin white arrow, septum; black thick arrow, newly accumulated outer layer; white thick arrow, old outer layer. Scale bars: 1 µm.

When colonies were treated with *n*-hexane for 15 min at room temperature, the cytoplasm retained its normal ultrastructure, but many holes appeared between the layers of the outer cell walls (Fig. 8A–C). The latter phenomenon was prominent in younger outer cell walls (Fig. 8B), but not in older ones (Fig. 8A). In contrast, the newly secreted oil droplets on the cell surface that were enclosed by a thin layer kept their unique droplet form under *n*-hexane treatment (Fig. 8A; right pair of cells, black **; 8C, right daughter cell, black **).

Then we treated colonies with cellulase RS and macerozyme for 16 h at 30°C, because the entire surface of a complex with cells and extracellular matrix is covered with the colony sheath (Fig. 2A1,2) that is composed of polysaccharides fibrils [29]. The outer cell walls were not affected, both for old layers and for younger layers just after lipid accumulation (Fig. 8D).

When the colonies were sequentially treated with *n*-hexane for 15 min followed by cellulase RS and macerozyme for 16 h, the ultrastructures of the cytoplasm except that in cells accumulating new oil droplets (Fig. 8F) were drastically damaged and the outer cell walls decomposed into thinner fibrils (Fig. 8E,F). The damage was more severe in younger outer cell walls (Fig. 8E,F, black thick arrow) than in older ones (Fig. 8E,F, white thick arrow). As was the case after treatment with only *n*-hexane, the newly secreted lipid droplets on the cell surface enclosed by a thin layer retained their form (Fig. 8F, black **), and the cytoplasm retained its normal ultrastructure (Fig. 8F), which may indicate that the thin layer provided some protection. In all these experiments, the cell walls surrounding the plasma membrane were not greatly damaged.

## Discussion

**Figure 9:**
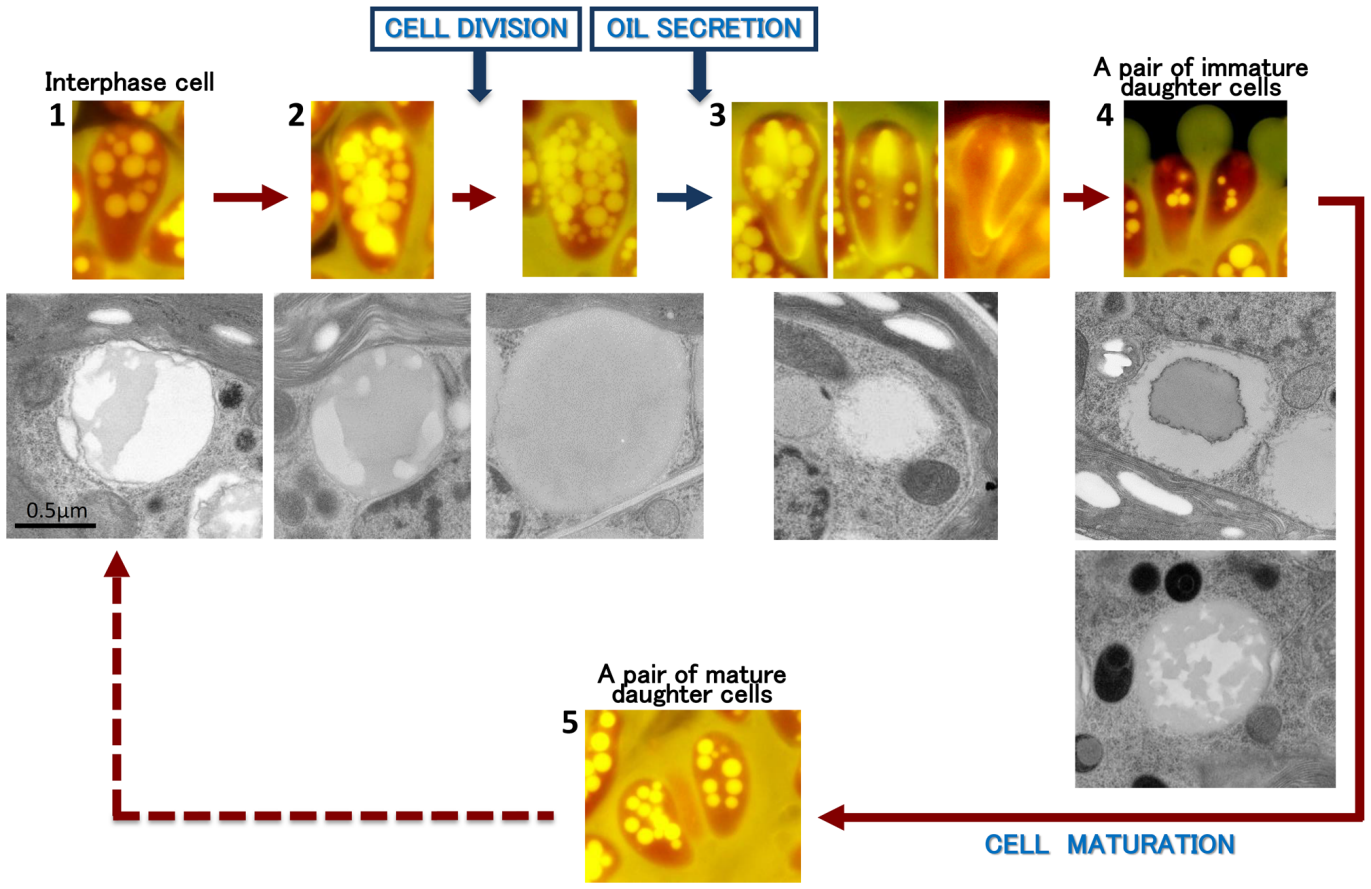
Transformation of lipid bodies during the cell cycle. Red solid line, increase in volume of lipid bodies; red dot line, keeping in volume of lipid bodies; blue solid line, decrease in volume of lipid bodies.

The accumulation of lipids in the extracellular space and transformation of lipid bodies in the cytoplasm during the cell cycle were clarified using fluorescence and electron microscopy. Figure 9 summarizes the results. In interphase cells (stage **1**), the lipid bodies occupy the space between the nucleus and the chloroplast. When the cells start to grow (stage **2** left), the sizes of the lipid bodies and of their electron-dense inclusions gradually increase. Just after septum formation (stage **2** right), the lipid bodies reach a maximum in their total volume and are fully filled with electron-dense inclusions. After the formation of the newer daughter cell wall and before the decomposition of the older mother cell wall (stage **3**), lipids appear between these two cell walls in the basolateral regions of the cells while lipid bodies disappear from the cytoplasm. Accompanying the size recovery of the chloroplasts in the daughter cells, the lipid bodies are reformed (stage **4**) and increase in number and size (stage **5**). In this cycle, the increase and decrease of the lipid bodies in the cytoplasm are strongly related to the accumulation of lipids at the cell surface.

### Role of the lipid bodies in *B. braunii*

The results of our study indicate that lipid bodies participate in the accumulation of extracellular lipids in both B. braunii race B in the present study as race A in our previous paper [28]. We are assuming that such direct involvement of lipid bodies in secretion of lipids is a novel finding, because lipid bodies have been generally considered to regulate the synthesis, utilization, and trafficking of intracellular lipids and play crucial roles in the cellular lipid metabolisms of various organisms, from bacteria to mammals and higher plants. Lipid bodies consist of a core of neutral lipids, which are predominantly triacylglycerol or cholesterol ester in mammalian cells [31]–[33] and triacylglycerol in the seeds of higher plants [34] [35]. The core is surrounded by a monolayer of phospholipids with associated proteins. Similarly, in green algae, lipid bodies isolated from Chlamydomonas reinhardtii [4] and Dunaliella salina [6], under nitrogen deprivation condition to induce accumulation of lipid bodies, contained 90% triacylglycerol plus 10% free fatty acids and 90% triacylglycerol plus <10% polar lipids, respectively. In contrast, the triacylglycerol content was predicted to be lower in B. braunii race B than in other algae [8]. In B. braunii race A, triacylglycerols account for 2 to 6% of total cell dry weight and 3 to 9% of total lipids [3], which suggests that the lipid bodies do not contain much triacylglycerols.

It is also noted that the fine ultrastructural changes during the maturation of lipid bodies are uniquely observed in B. braunii race B (Fig. 4,7) and race A [28]. They are quite different from the triacylglycerol-rich lipid bodies in rats [36] and in C. reinhardtii [5], in which the inclusion showed homogeneous electron density. In such organisms, their electron density of lipid bodies increased by the incorporation of newly synthesized triacylglycerols into the lipid bodies, induced by the addition of docosahexaenoic acid (DHA) or oleic acid in rat fibroblasts and induced by nitrogen deprivation in C. reinhardtii. Interestingly, when rat fibroblasts containing lipid bodies rich in methyl–β-cylodextrin-cholesterol complexes were treated with DHA, their lipid bodies changed to contain hazy zones or variously shaped zones with high and low electron density [36], which is similar to the lipid bodies in B. braunii in the present study. When these data are considered together, the lipid bodies of B. braunii appear to be special: they are not lipid-storage lipid bodies, but rather can be specialized to preparing lipids for secretion. Weiss et al. [37] found that in race B botryococcenes, the major hydrocarbon accumulating in the extracellular space, were also detected in the cytoplasmic lipid bodies by means of Raman spectroscopy, which suggests that the lipid bodies participate in the accumulation of extracellular lipids.

### Lipid body formation and transport pathway

The most popular current model of lipid body formation is that neutral lipids (lipid esters) are synthesized and deposited between the leaflets of the ER membrane, then bud as globules from the ER membrane to form independent lipid bodies that are bounded by a limiting monolayer of phospholipids and proteins associated with lipid bodies (see the reviews in [31]–[33] [38] [39]. This idea was first reported by Schwarzenbach [40] and elaborated on by Yatsu and Jacks [41], from the investigation of lipid formation in lipid-rich plant structures such as seeds and embryos. Supporting evidence was obtained by ultrastructural observations of lipid-accumulating cells from a variety of plant species prepared by means of conventional chemical fixation for electron microscopy [42]. On the other hand, a different process was reported by Robenek et al. using freeze-fracture electron microscopy that lipid droplet biosynthesis took place at specialized cup-shaped regions of the ER. The lipid droplet was cupped by closely apposed ER membrane outside the ER [43]. In *B. braunii* race B and race A [28], we have optimized the quick-freezing and freeze-substitution method to visualize the lipid bi-layer of the unit-membrane, but we could not obtain images that supported the popular model at the stage of lipid body formation before septum formation (Fig. 4E2) [28] and in race B during daughter cell maturation after lipid accumulation on the cell surface (Fig. 7D–F). In our analysis, lipid bodies were bounded by a limiting monolayer that was attached to the ER unit membrane (the lipid bi-layer; Fig. 7D) or covered by the ER sheet (Fig. 7E) as like the model by Robenek et al. [43]. One possible explanation of the difference comes from the different contents of the lipid bodies; high triacylglycerol contents in most previously examined cells, as discussed earlier, versus lower triacylglycerol contents in *B. braunii*. We also observed lipid bodies that seemed to be produced from the chloroplast surface (Fig. 7B,C), as was reported in the seeds [44] and the cotyledons [45] of *Sinapis alba*, and the green alga *C. reinhardtii* [46].

Among oleaginous microalgae, *B. braunii* is well known to produces especially large quantities of hydrocarbons and accumulates them in the extracellular space. In *B. braunii* race B, the biosynthetic pathways to produce botryococcenes and methylsqualenes have been clarified and several important enzymes that contribute to the production of these hydrocarbons have been identified [14]–[18]. However, there has been no discussion on the transport pathway of hydrocarbon precursors in the cytoplasm. It has been shown that the race B uses the MEP pathway to produce the universal precursors for isoprenoids and in higher plants, all enzymes in the MEP pathway have chloroplast signal sequences at their N termini. In this context, three distinct cDNA clones coding for isozymes of 1-deoxy-D-xylulose 5-phosphate synthase (DXS) that catalyzes the first step of the MEP pathway were isolated from the race B and all three turned out to be possessing chloroplast signal sequences at the N termini [18]. All other genes coding for enzymes in the MEP pathway cloned from the race B showed deduced amino acid sequences containing chloroplast signal sequences at their N termini (Okada, unpublished data). Thus, biosynthesis of the universal precursors for isoprenoids by *B. braunii* is expected to occur in the plastid as higher plants. On the other hand, the genes encoding key enzymes (SSL-1, 2 and 3) catalyzing the final steps from farnesyl diphosphate to C_30_ botryococcene (SSL-1 and 3) and squalene (SSL-1 and 2) have no chloroplast signal sequences [17], suggesting these reactions occur outside the chloroplast. In this study, we clarified that the rER was often in contact with both a chloroplast and lipid bodies in cells increasing lipid bodies and maturing lipid bodies (Fig. 7E,F), and the rER lost ribosomes from the surface facing the chloroplast (Fig. 7E,F) or lipid bodies (Fig. 4E2; 7D,E,F). These results may indicate that these contact sites are used for a channel through which precursors progressed from isoprenoids were transported from chloroplast to lipid bodies.

For the following migration from the lipid body to the cell surface, the ER would seem to be the most likely conduit. Because the cortical ER is especially prominent in *B. braunii* [28] [29], and it also lacks ribosomes from the surface facing the plasma membrane (Fig. 6E2) [29]. This speculation is fit the old data by a radiolabeling experiment [10], that race B does secrete cytoplasmic hydrocarbons. We also note that when the lipid bodies decreased they often attached to electron-dense spherical bodies enclosed by a unit membrane (Fig. 6A,B). Though the function of these electron dense bodies remains unknown, we like to suggest that they might be involved in processing inclusions of lipid bodies to be secreted as oil droplets in Figure 6.

### Extracellular lipid accumulation and colony development in B. braunii race B

Previous microscopical studies have shown that a colony of *B. braunii* is a botryoid organization of pyriform-shaped cells that are held together by a refringent matrix [19] [30] that contains lipids (Fig. 1B), and that colonies are composed of several daughter colonies (Fig. 1A) [29]. Recently, Weiss et al. studied the colony development of race B using quick-freeze deep-etch electron microscopy combined with biochemical and histochemical analysis [29]. They found that a colony was enclosed by a retaining wall festooned with a fibrillar sheath that was dominated by arabinose–galactose polysaccharides, which sequestered liquid hydrocarbons in the extracellular matrix.

Based on these previous reports and our present study, we propose a model of how a colony of race B develops. In our electron microscopical study, the extracellular matrix surrounding the basal part of the cells consists of outer cell walls that originated from successive cell divisions (Fig. 2B1), as was described by Metzger and Largeau [3]. In each division, lipids secreted after cell wall formation create multiple (≤6) layers of oil droplets arranged side by side at the basolateral region of the daughter cells (Fig. 6), then the oil droplets seem to fuse to create each continuous flat sheets that correspond in number to the number of original oil droplet layers. This lipid fusion might be triggered by collapse of the mother cell wall and thin layers at the cell apex. The combined flat lipid sheets at the basolateral regions from a pair of daughter cells remain during successive cellular division as cell-cell adhesion, and the colony gets larger with increase of the cell number (Fig. 2B1). At the same time, the flat lipid sheets that are rather flexible and only capturing the basolateral regions of the cells allow these increasing cells to be rearranged into a colony in which the apex of each cell is always positioned towards the colony's surface (Fig. 1; 2A,B; 3A,B). These groups of cells are further arranged in a spherical layer with individual cells connected via the multiple flat lipid sheets, like the flower stalks in a bouquet (Fig. 2A–C). A colony consists of several sub-colonies (Fig. 1), which may be produced by a series of rearrangements of cells at the colony surface to retain a unique colony size for each *B. braunii* strain. When the colony surface area exceeds a certain limiting area, then the mother colony divides into smaller daughter colonies, as the division manner proposed by Weiss et al. [29].

On the information of extracellular matrix constructing the colony, there is a big gap between the structure analyzed by electron microscopy and composition of extracted materials by chemical analysis [10] [11]. To bridge the gap, we tried to obtain ultrastructural information on certain chemical composition by treatment with reagents extracting lipids and/or enzymes degrading polysaccharides. The newly secreted oil droplets on the cell surface were enclosed by a thin layer, and retained their droplet form under treatment with *n*-hexane (Fig. 8C), and even with *n*-hexane followed by cellulase RS and macerozyme (Fig. 8F). The flat lipid sheets were stained with Nile red (Fig. 1) and were not digested by treatment with cellulase and macerozyme (Fig. 8D). On the other hand, *n*-hexane treatment for 15 min decomposed newly accumulated layers but not old ones (Fig. 8A–C). Based on these results, the newly secreted oil droplets appear to begin as a liquid and then some components in the droplets may start to polymerize to create the lipophilic flat sheets through acetal linkages of long chain fatty aldehydes and/or methyl squalene epoxides [47]. The liquid hydrocarbons may then accumulate in the lipophilic flat sheets. While degrees of polymerization of the polyacetals are still low in newly created lipophilic flat sheets, they would be susceptible to treatments with *n*-hexane and be degraded. On the other hand, they could become more resistant to such external reagents after sufficient polymerizations.

### Race B accumulate more lipids than race A

Our cytological examination showed that the ultrastructure of race A [28] and race B (the present study) were basically the same; both shared the similar location of organelles within a cell, the main extracellular lipid accumulation site at the basolateral region of the daughter cells, and the main stage for lipid accumulation after cell wall formation. Regarding the behavior of lipid bodies, the increase/decrease, size changes and inclusions of them are similarly seen as shown in Figure 9, stage 2 through stage 3. However, we notice the critical differences between two species as below:

1. Although race B has the lipid bodies in the interphase cells (Fig. 9, stage 1), two *B. braunii* strains belonging to race A (UTEX 2441 and 807/1) do not have lipid bodies at this stage. The formation of new lipid bodies in race B can occur during the maturation of the daughter cells (stage 4–5) as well as stage 2. In race A, new lipid bodies appear to be synthesized only during the early growing stage (stage 2).

2. During the extracellular lipid accumulation in the basolateral region of race A, lipid droplets on the cell surface form only one or two layers that is fewer than that of race B (≤6). This difference can explain the higher hydrocarbon content per unit cell dry weight in race B.

3. The extracellular lipid accumulation in the basolateral region occurs after recovery of the chloroplast volume in race A, but it starts earlier in race B before the chloroplast recovery completes (Fig 9, stage 4 through 5).

4. Lipid secretion from the cell apex has been shown in race A, but not prominent in race B. In race B, accumulation and maturation of precursors for a colony sheath was more prominent at cell apex (in preparation).

In addition, previous biochemical studies has characterized an important difference that race A synthesizes mainly alkadienes and alkatrienes [9] via the fatty acid synthetic pathway [48] but race B synthesizes mainly triterpenoids known as botryococcenes [10] [11] and methylsqualene [49] via the MEP pathway [50]. Thus, we consider that the expression and localization of key enzymes in each pathway are critical to understand how these differences are made. We are expecting that further studies on cellular and molecular bases on lipid bodies and accumulation of these *B. braunii* species will shed light on their unique hydrocarbon synthesis that will lead to use of these algae to be available resource of renewable energy.
